# In vitro toxicity assessment of graphene quantum dots using a 3D HepG2 model

**DOI:** 10.1007/s00204-026-04336-9

**Published:** 2026-03-10

**Authors:** Irma Durmišević, Anja Haverić, Sonja Žabkar, Alja Štern, Katja Kološa, Petra Jenuš Belec, Tamara Ćetković Pećar, Maida Hadžić Omanović, Sanjin Gutić, Iza Rozman, Sanin Haverić, Bojana Žegura

**Affiliations:** 1 Institute for Genetic Engineering and Biotechnology, University of Sarajevo, Zmaja od Bosne 8, Sarajevo, Bosnia and Herzegovina; 2 Department of Genetic Toxicology and Cancer Biology, National Institute of Biology, Večna pot 121, Ljubljana, Slovenia; 3 Biotechnical Faculty, University of Ljubljana, Jamnikarjeva 101, Ljubljana, Slovenia; 4 Department for Nanostructural Materials, Jožef Stefan Institute, Jamova cesta 39, Ljubljana, Slovenia; 5 Faculty of Science, University of Sarajevo, Zmaja od Bosne 33-35, Sarajevo, Bosnia and Herzegovina

**Keywords:** Nanomaterials, Graphene quantum dots, HepG2 spheroids, Toxicity

## Abstract

In the present study, two types of graphene quantum dots (GQDs) were investigated: green-emitting (G-GQDs) and blue-emitting (B-GQDs). Physicochemical characterisation was performed using transmission electron microscopy (TEM), zeta potential, and hydrodynamic radius measurements to evaluate the morphology, particle size, aggregation behaviour, and colloidal stability of the GQDs in both water and cell culture medium. G-GQDs exhibited superior colloidal stability and more uniform dispersion than B-GQDs, whereas both types showed reduced aggregation and surface charge in cell culture medium due to protein corona formation. Toxicological characterisation was performed using an in vitro human hepatocellular carcinoma (HepG2) 3D spheroid model, with GQDs exposures up to 250 µg/mL (100 µg/cm^2^). Cytotoxicity was measured using the CellTiter-Glo luminometric assay, while genotoxicity was evaluated by the comet assay and flow cytometric analysis of γH2AX and phosphorylated histone H3 (p-H3) after 24 h of exposure. Both GQDs induced dose-dependent cytotoxic effects in HepG2 spheroids. At non-cytotoxic concentrations, a dose-dependent increase in DNA damage was observed, as determined by the comet assay. However, no evidence of DNA double-strand breaks (γH2AX) or elevated p-H3 levels was detected, suggesting the absence of clastogenic and aneugenic activity. The observed DNA single-strand breaks may be partly attributed to reactive oxygen species induction. These results indicate that, although GQDs induced cytotoxicity and single-strand DNA damage, no clear evidence of more severe genotoxic effects was observed under the tested conditions. Further studies are warranted to elucidate underlying mechanisms and comprehensively assess the safety profile of GQDs for biomedical applications.

## Introduction

The rapid expansion of nanomaterial development over recent decades has led to increased human and environmental exposure, making the assessment of nanoparticle toxicity an essential step in identifying potential health and environmental hazards posed by these materials. Among the diverse classes of nanomaterials, graphene, a two-dimensional sheet of *sp*^2^-hybridised carbon atoms covalently bonded in a hexagonal lattice, has attracted significant scientific interest due to its remarkable electrical, mechanical, and optical properties (Kostarelos and Novoselov 2014). These exceptional features arise from the fact that carbon plays a fundamental role in the environment, being an integral component of all living organisms, and serves as a primary source of energy. However, it is important to emphasise that graphene-based nanomaterials exhibit considerable complexity and diversity in terms of their size, morphology, shape, surface charge, chemical composition, coating or surface modifications, synthesis methods, aggregation tendencies, and stability in biological media. The small size of GQDs, along with their physicochemical properties, plays a major role in determining their toxicity and biocompatibility. Generally, graphene quantum dots (GQDs) are characterized by favourable biocompatibility, and low toxicity (Nasrollahi et al. 2019). Accordingly, being commercially available, they are highly promising for applications in research and pre-clinical diagnostics (Ghanbari et al. 2017; Chauhan et al. 2017), drug delivery (Iannazzo et al. 2017) and bioimaging (Kuo et al. 2020). Factors such as dose, route, and duration of exposure, as well as cell type, entry pathways, tissue distribution, excretion, and specific patterns and sites of cellular uptake, further modulate their biological activity (Ou et al. 2016). As a result, these nanomaterials may exert detrimental effects on cells through various mechanisms. Particular attention should therefore be paid to their potential (geno)toxic effects (Liao et al. 2018; Papanikolaou et al. 2023). Zero-dimensional GQDshave recently emerged as notable members of the carbon nanomaterial family. GQDs are nano-sized fragments of graphene sheets, typically with a lateral dimension below 100 nm (Novoselov et al. 2004; Shen et al. 2012). Their large surface area, together with the presence of hydrophilic groups at their edges, makes them highly suitable for surface functionalization and chemical modification (Shen et al. 2012; Li et al. 2017; Havanur et al. 2019). Owing to their exceptional physicochemical properties, GQDs have great potential for applications in optics, electrochemistry, and biomedicine, including drug delivery (Shen et al. 2012; Havanur et al. 2019; Kumar et al. 2020). They are also considered highly versatile materials with promising applications in sensing, energy conversion, nanomedicine, single-electron transistors, spintronics, memory, and more (Wang et al. 2016). However, when considering the use of graphene-based nanomaterials, including GQDs, particularly for biomedical purposes, it is essential to ensure that they are biocompatible and do not exhibit toxic effects, despite of the large improvement in the biocompatibility. Understanding the interactions of graphene-based nanomaterials within biological systems, tissues, and cells, along with the systematic studies on their potential long-term toxicity, is crucial for their biosafety evaluation, which is necessary to ensure their safe application in biomedical fields (Zhao et al. 2020). The potential toxic mechanisms of graphene family nanomaterials (GFNs) include inflammatory response, necrosis, apoptosis, DNA damage, and autophagy, among others (Ou et al. 2016). However, the reported toxic effects of GQDs in the literature remain conflicting. Several studies indicate that GQDs exhibit good biocompatibility (Yuan et al. 2014; Singh et al. 2019; Qu et al. 2019) and low cytotoxicity in primary human CD34^+^ haematopoietic stem cells (HSCs) (Fasbender et al. 2019), as well as in human cervical cancer (HeLa) and lung epithelial (A549) cell lines (Chong et al. 2014), suggesting that small-sized GQDs do not disrupt or damage lipid membranes in these cells (Liang et al. 2020). In contrast, a study conducted on human breast cancer (MCF-7) cells reported significant cytotoxicity (Murugan et al. 2017), indicating that GQDs toxicity may be highly dependent on the specific cell type and experimental conditions.

This study aimed to evaluate the toxicological profile of two commercially available graphene quantum dots, blue (B-GQDs) and green (G-GQDs), which share the same particle size but differ in their band gaps, using an in vitro 3D HepG2 cell model. The 3D cell model was selected because it offers a more accurate representation of the in vivo cellular microenvironment compared to conventional 2D models, thereby providing more physiologically relevant insights into cellular responses. Cytotoxicity of the GQDs was initially assessed using an ATP-based assay, followed by genotoxicity evaluation at non-cytotoxic concentrations using the comet assay. To further assess potential clastogenic and aneugenic effects, γH2AX- and pH3-positive cells were quantified by flow cytometry. This comprehensive approach is essential for the assessment of the safety and bioactivity of GQDs.

## Materials and methods

### Chemicals

Minimum essential medium eagle (MEME), penicillin/streptomycin, Na-pyruvate, non-essential amino acids (NEAA), NaHCO_3_, L-glutamine, dimethylsulphoxide (DMSO), methylcellulose, benzo(a) pyrene (B(a)P, colchicine, formaldehyde, blue (B-GQDs; 900726-50MG) and green graphene quantum dots (G-GQDs; 900713-50MG) powder were purchased from Sigma (St. Louis, MO, USA), while etoposide (ET) was obtained from Santa Cruz Biotechnology (Santa Cruz, CA, USA). Low melting point agarose (LMP), normal melting point agarose (NMP), trypsin-EDTA (0.25%), foetal bovine serum (FBS), and TrypLE™ Express Enzyme were obtained from Gibco (Thermo Fisher Scientific, Grand Island, NY, USA), and Hoechst 33258 dye was from Invitrogen (Waltham, MA, USA). Phosphate-buffered saline (PBS), methanol, and ethanol were purchased from PAA Laboratories (Dartmouth, NH, USA). H2AX pS139 APC Antibody (130-123-256), Histone H3 pS28 PE Antibody (130-124-883), REA Control Antibody (I), human IgG REAfinity™ APC (130-120-709) and PE (130-118-347) were obtained from Miltenyi Biotec (Bergisch Gladbach, Germany), and CellTiter 96^®^AQueous cell proliferation assay [3-(4,5-dimethylthiazol-2-yl)-2,5-diphenyltetrazolium bromide; MTS] was from Promega (Madison, WI, USA) and GelRed from Biotium (Fremont, CA, USA).

### B-GQDs and G-GQDs

Graphene quantum dots (GQDs) used in this study are commercially available from Sigma (St. Louis, MO, USA). They were synthesized by treating coal with hot concentrated nitric acid. The two types of quantum dots obtained through this method share very similar chemical and physical structures. The disk-shaped particles exhibit a topographic height between 1 and 2 nm, corresponding to a few graphene layers stacked in a graphitic structure, with a lateral diameter of less than 5 nm (Sigma-Aldrich 2025). The main difference in physical properties between the two nanomaterials lies in their photoluminescence spectrum: G-GQDs emit light with the maximum intensity at 525 nm, whereas blue-emitting GQDs (B-GQDs) peak at 445 nm. This difference in fluorescence may reflect subtle variations in chemical composition (i.e., presence or absence of oxygen and nitrogen functional groups). A summary of physical properties for the two studied GQDs is presented in Table 1.

**Table 1:** Physicochemical properties of two types of graphene quantum dots

Physical and chemical properties	B-GQDs	G-GQDs
Form	Powder	Powder
Colour	Brown	Black
Odor	Odourless	Odourless
Topographic height	1–2.0 nm	1–2.0 nm
Particle size/diameter	< 5 nm	< 5 nm
Excitation (λ_ex_)	350 nm	485 nm
Emission (λ_em_)	445 nm ± 10 nm	525 nm
Melting point/freezing point	N/A	> 400 °C
pH	6.0–9 at 40 g/l at 25 °	6.0–9 at 40 g/l at 25 °C
Vapor pressure	< 0.01 hPa at 20 °C	< 0.01 hPa at 20 °C
Density	0.250–0.600 g/cm3	0.250–0.600 g/cm3
Bulk density	250–550 kg/m3 at 20 °C	250–550 kg/m3 at 20 °C

Information adapted from Sigma-Aldrich (2025). Source: Safety data sheet G-GQDs https://www.sigmaaldrich.com/BA/en/product/aldrich/900713; B-GQDs https://www.sigmaaldrich.com/BA/en/product/aldrich/900726; accessed 30 November 2025

### Structural and physicochemical properties

Transmission electron microscopy (TEM) of B-GQDs and G-GQDs was performed using a Jeol 2100 instrument at an accelerating voltage of 200 kV. For TEM measurements, a small amount of B-GQDs or G-GQDs was dispersed in deionised water and drop-deposited onto a holey carbon-coated copper TEM grid. Zeta potential and hydrodynamic radius of B-GQDs and G-GQDs were measured by Litesizer DIF 500 (Anton Paar GmbH). Both quantities were assessed in deionised water and in cell growth medium at room temperature, with the pH recorded for each measurement. For each sample, ten consecutive measurements were carried out, and the results are presented as the mean of these measurements.

### Cell culture and 3D cell model (spheroid) formation

HepG2 cells (ATCC, HB-8065™) were cultured in MEME supplemented with 10% FBS, 100 IU/mL penicillin/streptomycin, 1% NEAA, 0.1 g/mL sodium pyruvate, 0.1 g/mL sodium bicarbonate, and 2 mM L-glutamine at 37 °C in a humidified 5% CO_2_ atmosphere. HepG2 spheroids were formed using the forced-floating method in 96-well U-bottom low-attachment plates (Falcon, Corning Incorporated, Corning, NY, USA) with growth medium containing 4% methylcellulose, following the protocol by Štampar et al. (2019). An initial density of 3000 cells per spheroid was used. Plates were centrifuged for 1.5 h at 28 °C and 850 *g* to aggregate the cells into spheroids, which were subsequently cultured for 72 h at 37 °C under static conditions in 5% CO_2_ humidified atmosphere. After 3 days, the growth medium was removed, and the spheroids were exposed to various concentrations of the studied GQDs for 24 h. All experiments included a vehicle (0.2% MilliQ), a negative (cell culure medium), and assay-specific positive controls (PC). Spheroid growth and morphology were regularly monitored using a Ti Eclipse inverted microscope (Nikon, Tokyo, Japan).

### Cytotoxicity testing—ATP assay

The viability of spheroids was assessed using the CellTiter-Glo^®^ 2.0 Assay following 24 h of exposure. Spheroids were treated with B- and G-GQD at concentrations of 2, 10, 50, 100, and 250 µg/mL (corresponding to 0.8, 4, 20, 40, and 100 µg/cm^2^). DMSO (15%) was used as a positive control. For each experimental point, five spheroids per concentration were used, each representing an independent replicate. They were transferred from the U-bottom culture plate to a white-opaque 96-well plate (Corning Incorporated, Corning, NY, USA), each well containing 50 µL of medium. Subsequently, 50 µL of CellTiter-Glo reagent was added to each well and mixed until the spheroids were fully disintegrated. The plates were then incubated for 10 min, and viability was measured using a luminometer (Synergy HTX, BioTek, Winooski, VT, USA). All experiments were performed independently three times.

### Genotoxicity testing—Comet assay

Single-cell suspensions from spheroids were obtained using a combination of mechanical disruption and enzymatic digestion (Štampar et al. 2019). Following 24-hour exposure to B- and G-GQDs at concentrations of 12.5, 25, 50, and 100 µg/mL (corresponding to 5, 10, 20, and 40 µg/cm^2^) and 30 µg/mL BaP as the positive control, six spheroids per concentration were collected and treated with a mixture of collagenase and TrypLE, diluted in serum-free medium (1:20:9), for 10 min. Spheroids were then mechanically dissociated into a single-cell suspension by pipetting. The subsequent steps followed the standard monolayer cell culture protocol. Briefly, 30 µL of the cell suspension was mixed with 70 µL of 1% low-melting-point (LMP) agarose and applied to fully frosted slides pre-coated with a layer of 1% normal-melting-point (NMP) agarose. The slides were lysed in a solution containing 0.1 M EDTA, 2.5 M NaOH (pH 10), 0.01 M Tris, and 1% Triton X-100 for 1 h at 4 °C. DNA was unwound and electrophoresed in an alkaline solution (300 mM NaOH, 1 mM EDTA, pH 13) for 20 min at 25 V and 300 mA (0.5–1 V/cm). The slides were then neutralised using 0.4 M Tris buffer (pH 7.5), and the gels were stained with GelRed. Images were captured and analysed using an Eclipse 800 fluorescence microscope (Nikon, Tokyo, Japan) equipped with a Basler camera and the Comet IV image analysis software (Perceptive Instruments Ltd., Haverhill, United Kingdom). Three independent experiments were conducted, with 50 randomly selected nuclei analysed per experimental condition. Results were expressed as the percentage of tail DNA.

### Clastogenic and aneugenic activity—γH2AX and pH3-positive cells

HepG2 spheroids were exposed to graded concentrations of B- and G-GQDs (12.5, 25, 50, and 100 µg/mL; corresponding to 5, 10, 20, and 40 µg/cm^2^) for 24 h, respectively. Etoposide (ET, 1 µg/mL) and colchicine (0.04 µg/mL) were used as positive controls for 24 h for the detection of γH2AX and pH3-positive cells, respectively. Spheroids were dissociated as described in Sect.  “Genotoxicity testing—Comet assay”. Single-cell suspensions were washed twice with 1× PBS and fixed in 4% PFA. For flow cytometric analysis, cells were labelled with anti-H2AX pS139 antibody to detect DNA double-strand breaks (DSB) and anti-histone H3 pS128 antibody to assess aneugenic activity as described previously (Sendra et al. 2023; Štern et al. 2024). REA-APC and REA-PE controls were applied to exclude non-specific antibody binding. For each concentration, 24 spheroids were collected across three biologically independent experiments were conducted, and 10,000 events were acquired for each measured sample using the flow cytometer MACSQuant Analyzer 10 and MACSQuantify™ software (Miltenyi Biotec, Bergisch Gladbach, Germany). Raw data were exported from the MACSQuantify software and analysed with FlowJo V10 software (BD, Franklin Lakes, NJ, USA).

### Statistical analysis

Statistical analyses of the toxicological data were performed using GraphPad Prism version 10 (GraphPad Software), with *p* < 0.05 considered statistically significant. Differences in cell viability between B- and G-GQDs exposed spheroids and controls were assessed using one-way ANOVA followed by Dunnett’s multiple comparison test. For comet assay results, the non-parametric Kruskal-Wallis test followed by Dunn’s multiple comparison test was applied. Statistically significant differences in the number of pH3-positive cells between exposed and control groups were evaluated using one-way ANOVA with Dunnett’s multiple comparison test. Differences in the intensity of APC (γH2AX) fluorescence signal were analysed using two-way ANOVA followed by uncorrected Fisher’s LSD test.

## Results and discussion

Graphene quantum dots (GQDs) are a class of carbon-based nanomaterials with unique physicochemical properties, including excellent biocompatibility, fluorescence, and tuneable electronic characteristics. As their applications continue to expand, particularly in biomedicine, bioimaging, and drug delivery, concerns have emerged regarding their potential toxicity and long-term safety. Given their small size, high surface reactivity, and ability to interact with biological macromolecules, it is essential to assess their potential adverse effects, including genotoxicity and other cellular stress-related responses. The physicochemical properties of GQDs, such as surface charge, functionalization, and fluorescence characteristics, can modulate interactions with cells and biomolecules, influencing biological outcomes and toxicity. Therefore, comprehensive *in vitro* testing strategies are necessary to evaluate their biological impact, particularly in physiologically relevant models.

Conventional cytotoxicity and genotoxicity studies often rely on two-dimensional (2D) cell cultures, which, despite their widespread use, fail to replicate the complexity of the *in vivo* tissue environment (Edmondson et al. 2014; Du et al. 2020). In contrast, three-dimensional (3D) cell culture models, such as hepatic spheroids, provide a more physiologically relevant system by better mimicking key aspects of liver tissue architecture, including cell-cell and cell-matrix interactions, oxygen and nutrient gradients, and metabolic activity (Shah et al. 2018; Štampar et al. 2021, 2024). Given that the liver is a key organ involved in the clearance and accumulation of nanoparticles, 3D liver models are particularly valuable for evaluating their potential toxicological effects. Recently, Varet et al. (2024) demonstrated that, compared to 2D cultures, 3D hepatic models are more effective for detecting genotoxic effects of nanomaterials. Building on these findings, the use of advanced in vitro models allows for a more accurate assessment of GQDs-induced cellular responses, particularly DNA damage, thereby supporting the Safe-by-Design approach and application of these nanomaterials.

### Structural and physicochemical properties of B-GQDs and G-GQDs

**Fig. 1:**
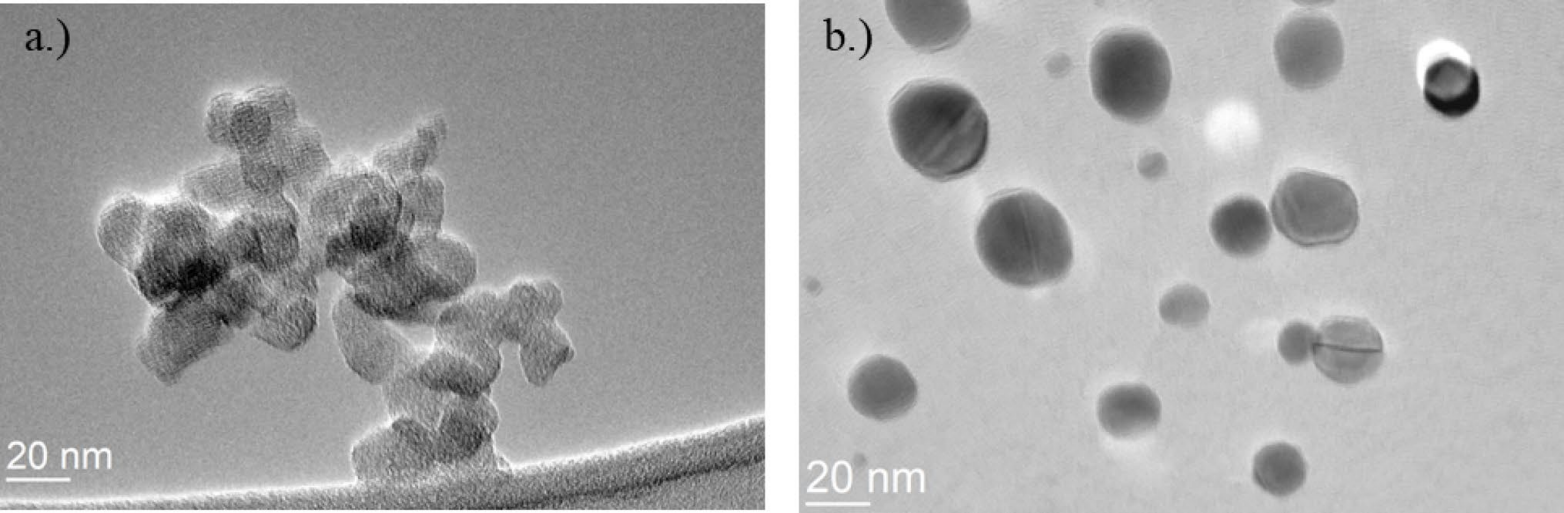
Bright-field TEM images of **a** blue (B-GQDs) and **b** green (G-GQDs) graphene quantum dots, illustrating differences in shape, dispersion, and aggregation behaviour

A comprehensive characterisation of the structural and physicochemical properties of GQDs is essential to understand their stability, dispersion behaviour, and potential biological interactions. In this study, we assessed key parameters including transmission electron microscopy, zeta potential, and hydrodynamic radius, for both green (G-GQDs) and blue (B-GQDs) graphene quantum dots to evaluate their colloidal stability, and surface charge under biologically relevant conditions. Transmission electron microscopy (TEM) analysis of B- and G-GQDs revealed distinct morphological differences between the two types of particles (Fig. 1). B-GQDs appeared more irregularly shaped and tended to form aggregates (Fig. 1a), while G-GQDs were predominantly spherical to slightly elliptical and exhibited uniform dispersion (Fig. 1b). The particle size of B-GQDs was approximately 20 nm, while G-GQDs displayed a bimodal size distribution, with one population below 10 nm and another around 20 nm.

**Fig. 2:**
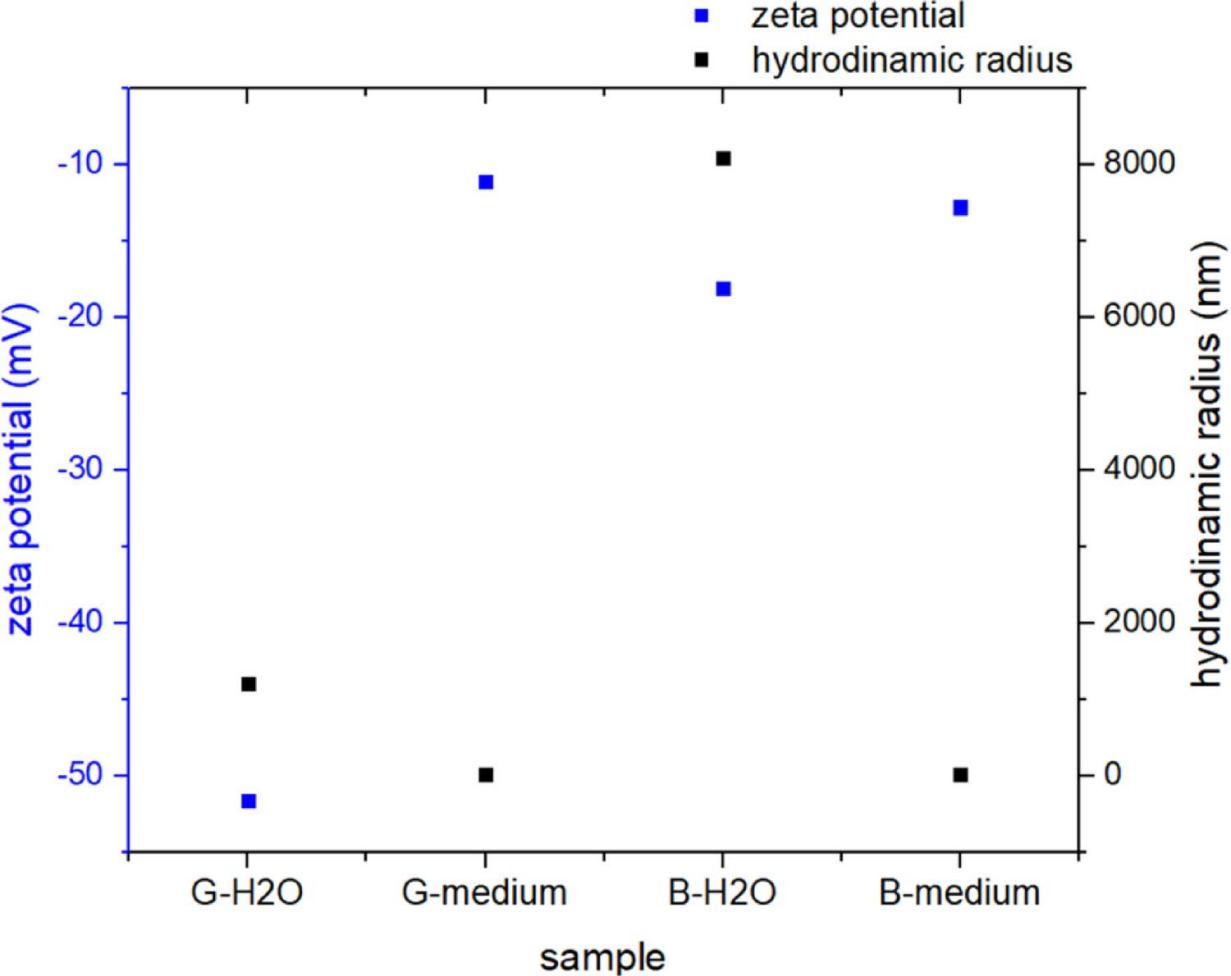
Zeta potential (mV, blue squares) and hydrodynamic radius (nm, black squares) of blue (B-GQDs) and green (G-GQDs) graphene quantum dots measured in water (H_2_O) and cell culture medium. G-GQDs display a higher negative zeta potential and smaller hydrodynamic radius in water compared to B-GQDs, indicating better colloidal stability. In medium, both samples show reduced zeta potential and hydrodynamic radius, consistent with ionic screening and protein corona formation. The data illustrate differences in surface charge and colloidal behaviour under biologically relevant conditions

The zeta potential and hydrodynamic radius of B-GQDs and G-GQDs were determined in both water and cell culture medium (Fig. 2). In water, both samples exhibited a higher negative zeta potential (G-GQDs-H_2_O: − 51.6 mV, B-GQDs-H_2_O: − 18.1 mV), indicating stronger electrostatic repulsion, which generally suggests good colloidal stability. In contrast, the zeta potential values decreased significantly in the cell culture medium (B-GQDs medium: − 12.8 mV, G-GQDs medium: − 11.1 mV), likely due to electrostatic screening by ions and adsorption of proteins from the medium (e.g., serum proteins binding to the quantum dots), leading to the formation of a protein corona on the quantum dots.

In water, the hydrodynamic radius was much larger for both samples (B-GQDs-H_2_O: 8086.1 nm, G-GQDs-H_2_O: 1209.4 nm), possibly due to quantum dots aggregation. This observation is consistent with the TEM analysis (Fig. 1), which shows a clear aggregation of B-GQDs in water. In contrast, when suspended in cell culture medium, the hydrodynamic radius markedly decreases (B-GQDs-medium: 25.5 nm, G-GQDs-medium: 27.3 nm), likely due to the formation of a protein corona that stabilises the quantum dot particles, preventing large aggregates. The hydrodynamic radius determined in cell medium also agrees well with the TEM observations of B-GQDs and G-GQDs particle sizes, further supporting the role of medium components in modulating GQDs colloidal behaviour. The G-GQDs sample showed a much higher absolute zeta potential in water compared to the B-GQDs sample, indicating better colloidal stability in its pure form. In contrast, the B-GQDs sample exhibited pronounced aggregation in water, with a hydrodynamic radius of 8000 nm, suggesting a strong tendency to form large aggregates when not stabilised. When dispersed in cell culture medium, both samples showed reduced hydrodynamic radii, consistent with adsorption of biomolecules such as serum proteins, which likely contribute to colloidal stabilisation. The decrease in zeta potential and hydrodynamic radius in the medium is expected due to high ionic strength and complex interactions between biomolecules.

### Toxicological characterisation of B-GQDs and G-GQDs

HepG2 cells are among the most widely used models in genetic toxicology due to their expression of the wild-type tumour suppressor p53 (Bressac et al. 1990), making them well-suited for studying p53-regulated responses to DNA damage. Three-dimensional (3D) hepatic spheroids derived from HepG2 cells offer an advanced model that better recapitulates key features of liver tissue architecture such as cell–cell and cell–matrix interactions, oxygen and nutrient gradients, and metabolic activity, compared to traditional two-dimensional (2D) cultures. Culturing HepG2 cells as 3D spheroids therefore enhances the model by better replicating  in vivo cell morphology and physiology, enabling the generation of more accurate and predictive data. This improvement is crucial for the safety assessment of human exposure to a wide range of environmental and chemical agents, including nanomaterials. Using HepG2 spheroids as an advanced in vitro model allows for a more precise evaluation of GQDs-induced cellular responses, particularly DNA damage and oxidative stress, thereby contributing to the safer design and application of these nanomaterials.

**Fig. 3:**
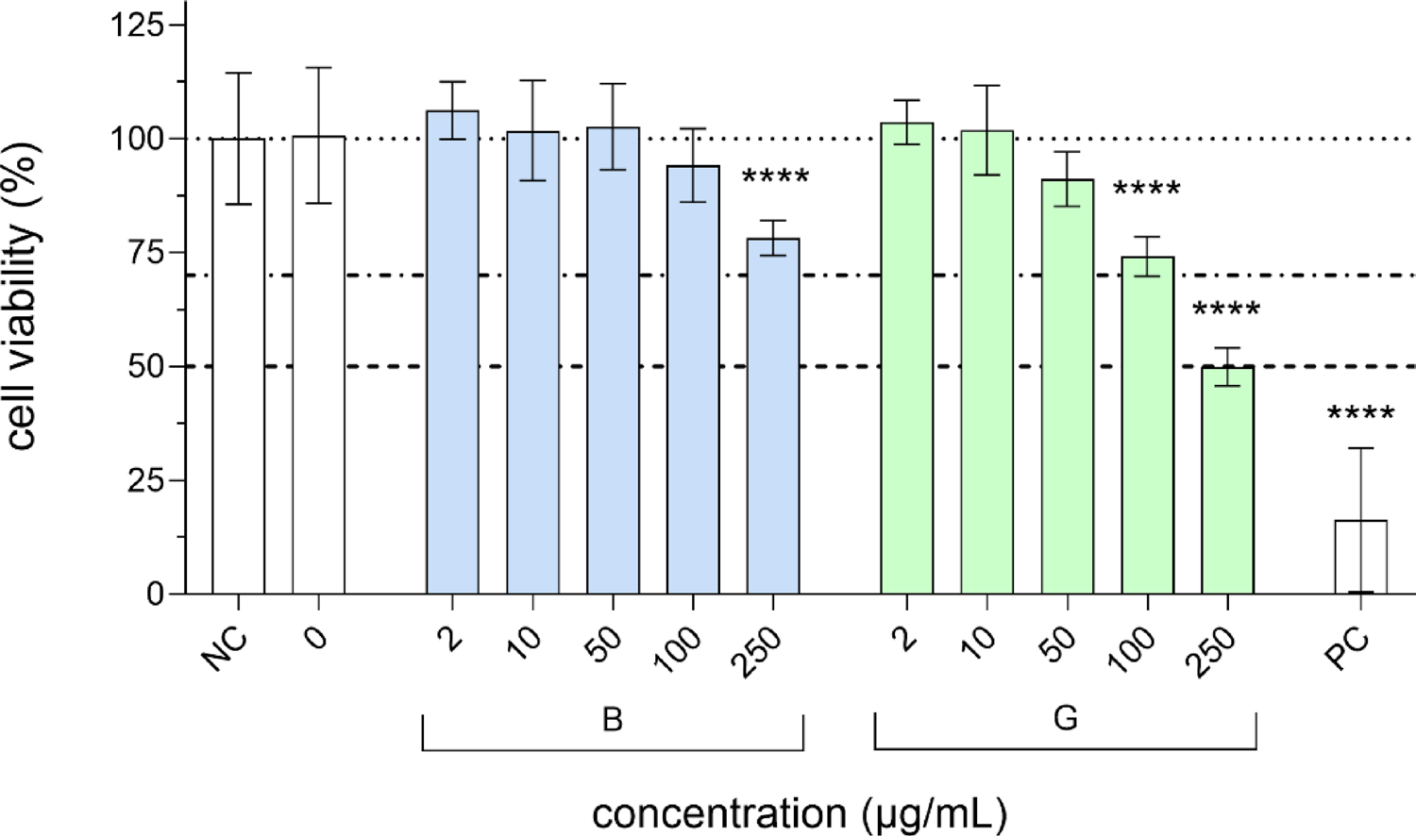
Viability of HepG2 cells in spheroids after 24-hour exposure to blue (B-GQDs) and green (G-GQDs) graphene quantum dots. Cytotoxicity was determined using the CellTiter-Glo^®^ 2.0 Cell Viability Assay. Cell viability is expressed as % of the solvent control − 0 (0.2% MilliQ). NC is the negative control (cell growth media), PC is the positive control (15% DMSO). The dashed lines (top to bottom) represent the 100%, 70% and 50% viability thresholds. Asterisks (*) indicate a statistically significant difference between GQDs-exposed cells and the solvent control (**** *p* ≤ 0.0001)

The cytotoxic effects of B- and G-GQDs in HepG2 spheroids were evaluated using the CellTiter-Glo^®^ ATP assay at GQDs concentrations of 0, 2, 10, 50, 100, and 250 µg/mL, corresponding to 0.8, 4, 20, 40, and 100 µg/cm^2^. These concentrations were selected based on the OECD Study Report and Preliminary Guidance on the Adaptation of the In Vitro Micronucleus Assay (OECD TG 487) for Testing of Manufactured Nanomaterials (OECD 2022). Our results (Fig. 3) show that B-GQDs exhibited pronounced cytotoxicity only at 250 µg/mL, where cell viability decreased to approximately 75%. In contrast, G-GQDs demonstrated stronger cytotoxicity at both 100 µg/mL and 250 µg/mL, with cell viability decreasing to approximately 50% at 250 µg/mL, indicating a higher overall cytotoxic activity of G-GQDs (Fig. 3). Notably, the cytotoxicity was observed at concentrations higher than those typically suggested by the OECD guidelines. These results are consistent with literature data showing that most studies report a typical ‘cut-off’ concentration of approximately 200 µg/mL, below which high cell viability, generally exceeding 70%, is usually observed (Şenel et al. 2019; Liang et al. 2020; Goldstein et al. 2023). It is well established in the literature that the toxicity of GQDs varies considerably depending on the particle size and the functional groups present on their surface. Not only can the presence of different surface modifications and functional groups contribute to toxicity, but variations in their relative abundance can also cause serious toxicity concerns (Wang et al. 2016; Lou et al. 2024).

**Fig. 4:**
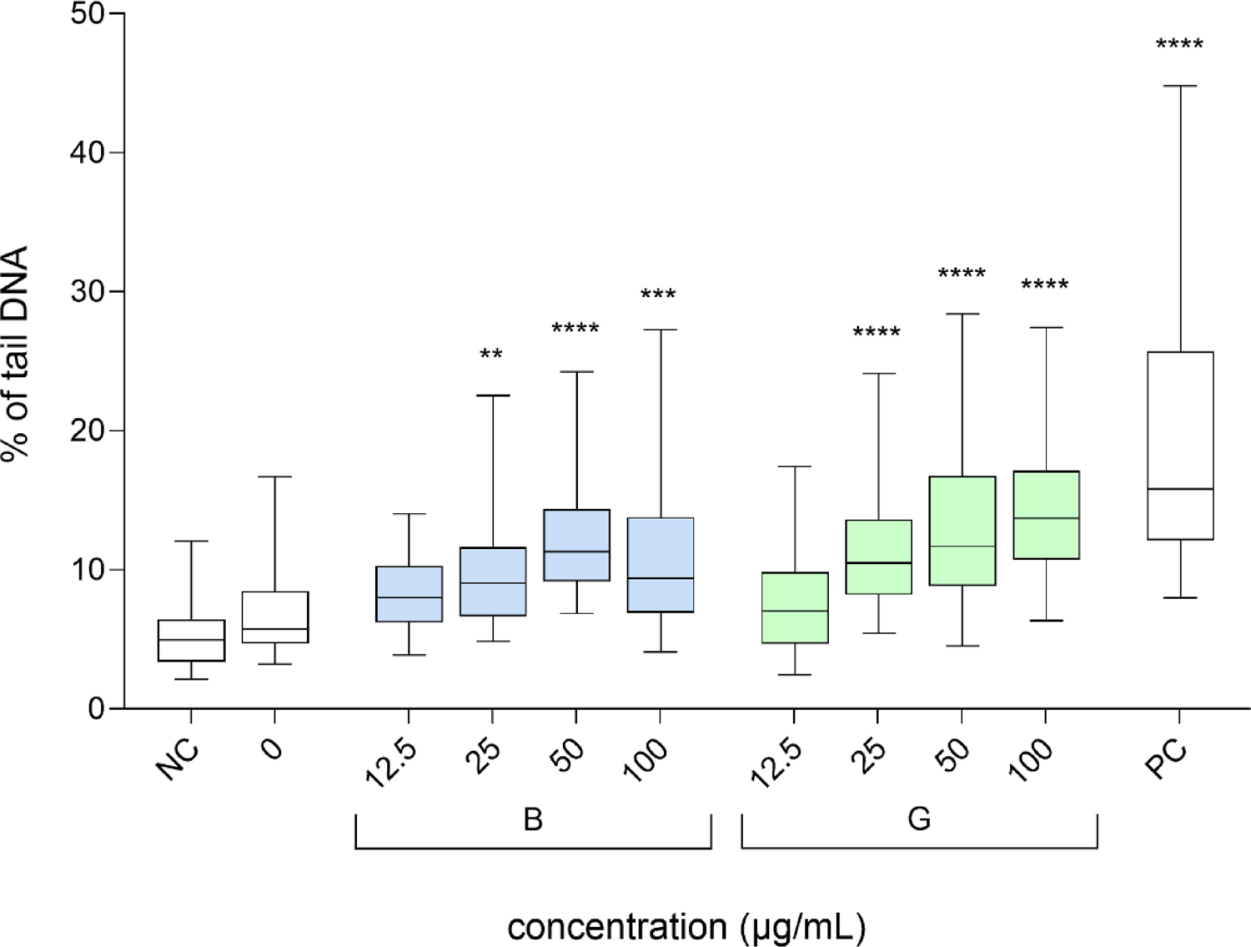
Induction of DNA damage in HepG2 spheroids after 24-hour exposure to blue (B-GQDs) and green (G-GQDs) graphene quantum dots. Data are expressed as % of DNA in the “comet tail” and presented as quantile box plots (95% confidence interval). NC is the negative control (growth medium), 0 is the solvent control (0.2% MilliQ), and PC is the positive control (30 µg/mL BaP). Asterisks (*) indicate a statistically significant difference (Kruskal–Wallis nonparametric test and Dunn’s multiple comparison test) between the solvent control and the exposed cells (** *p* ≤ 0.01, *** *p* ≤ 0.001, **** *p* ≤ 0.0001)

Furthermore, the genotoxic activity of B-GQDs and G-GQDs in HepG2 spheroids was assessed using the comet assay, γH2AX, and pH3 assays following a 24-hour exposure to non-cytotoxic concentrations (Azqueta et al. 2022). Both GQDs, induced a significant, dose-dependent increase in the formation of DNA strand breaks, at concentrations above 25 µg/mL, as detected by the comet assay (Fig. 4). The comet assay is a sensitive method for detecting DNA damage, including DNA strand breaks and other lesions that are converted into strand breaks under alkaline conditions. It can also identify DNA repair activity by revealing the presence of DNA single-strand breaks (SSBs) and double-strand breaks (DSBs), along with alkali-labile sites, such as apurinic/apyrimidinic sites. Additionally, the assay detects DNA-DNA and DNA-protein cross-links, oxidised and alkylated nucleobases, UV-induced cyclobutane pyrimidine dimers, and certain chemically induced DNA adducts. It also captures transient SSBs that arise during DNA damage repair (Møller et al. 2020). Although not yet a formally validated method, the in vitro comet assay is widely used in nanoparticle research to assess genotoxicity (Azqueta and Dusinska 2015). Its broad applicability provides insights into genotoxic mechanisms and helps clarify the relationship between nanoparticle physicochemical properties and their potential to induce DNA damage. Given its sensitivity and relevance, it is commonly applied in various fields, including biomedicine, where nanoparticles are explored for drug delivery, imaging, and regenerative medicine. The comet assay is also recommended by EFSA scientific committee (2021) for the risk assessment of nanomaterials in food and feed.

The results of the comet assay revealed a concentration-dependent increase in DNA strand breaks following exposure to both B-GQDs and G-GQDs in HepG2 spheroids (Fig. 4). DNA damage was statistically significantly elevated at concentrations ≥ 25 µg/mL for both types of GQDs. B-GQDs exposure led to a slightly higher increase in tail intensity than G-GQDs at lower concentrations, suggesting a marginally stronger genotoxic effect under these conditions. This observation may be related to their more irregular shape and pronounced aggregation behaviour of B-GQDs, as shown by TEM and Dynamic Light Scattering (zeta potential and hydrodynamic radius) analyses, which could enhance interactions with cellular components or impair DNA repair mechanisms. In addition, the observed DNA damage could be linked to elevated reactive oxygen species (ROS) levels following GQDs exposure, potentially leading to oxidative DNA damage and transient lesions due to increased DNA repair activity. Although direct measurements of oxidative stress were not performed in this study, it is plausible that mild oxidative stress contributes to the genotoxic effects. However, other potential mechanisms may also be involved, including direct interaction with DNA or histones, interference with DNA replication or repair, lipid peroxidation, or secondary genotoxicity mediated by inflammation-induced downstream effects (Evans et al. 2019).

Among various types of DNA damage, DSBs are considered the most severe, as they pose a major challenge for repair. If not properly repaired, they can lead to genomic instability, mutations and ultimately contribute to carcinogenesis (Scully and Xie 2013). A key early response to DSBs is the rapid phosphorylation of histone H2AX at serine 139 (γH2AX), which accumulates at DSB sites, forming foci that directly correlate with the number of DSBs, making γH2AX a well-established and highly sensitive biomarker for DSB induction and clastogenicity (Mah et al. 2010; Valdiglesias et al. 2013). While γH2AX serves as a marker of clastogenic activity, phosphorylated histone H3 (p-H3) is an indicator of aneugenic effects (Khoury et al. 2016). Unlike clastogens, which induce direct DNA damage, aneugens interfere with non-DNA targets such as spindle kinases and microtubule fibres, disrupting key processes in cell division and leading to improper chromosome segregation during mitosis. Such disruption can result in mitotic arrest or abnormalities in the number and morphology of mitotic cells. Since phosphorylation of histone H3 is a reliable biomarker for mitotic cells, it is also considered a marker for aneugenic potential (Lynch et al. 2019).

**Fig. 5:**
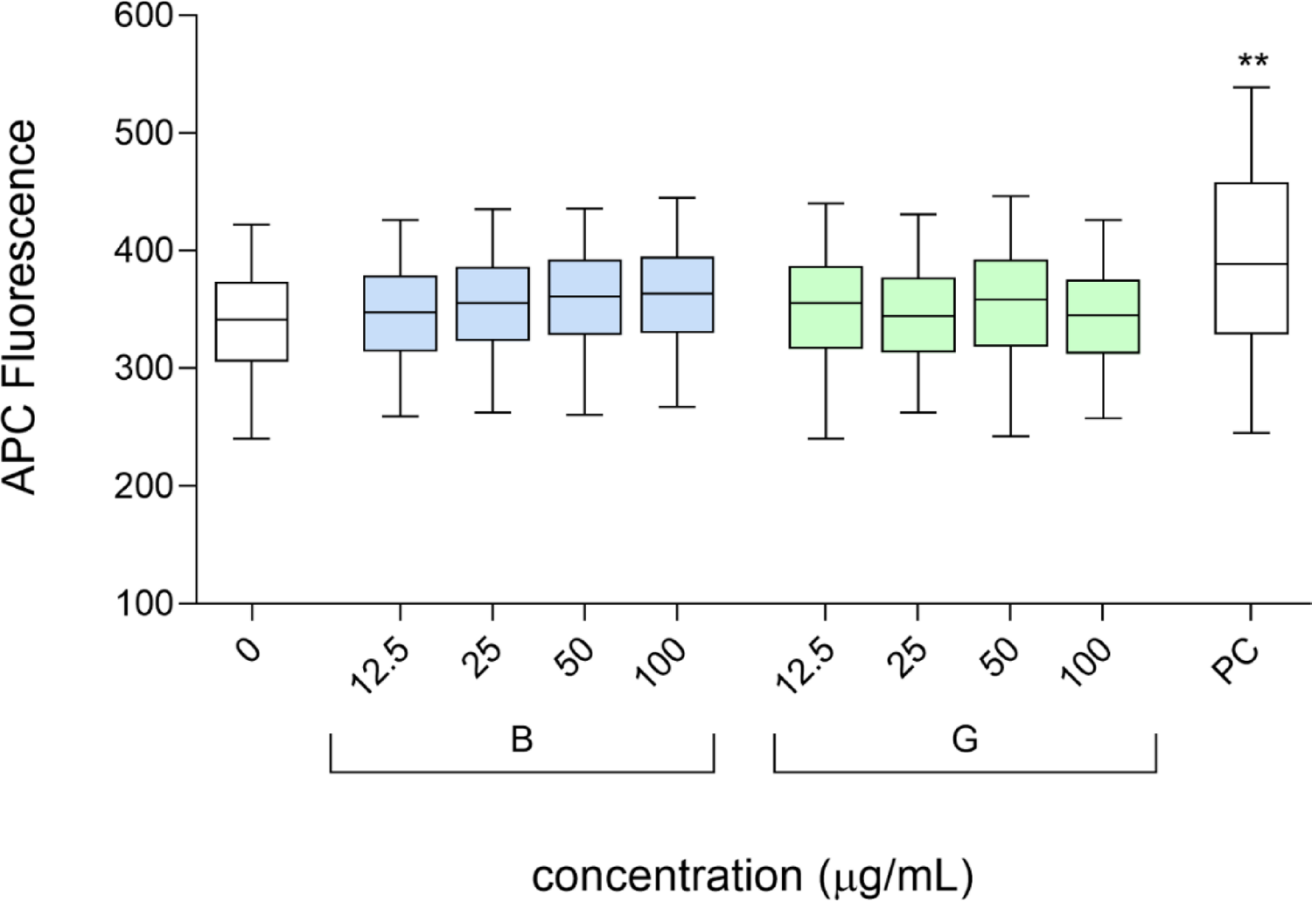
Induction of DNA double-strand breaks (DSBs) in HepG2 cells after 24-hour exposure to blue (B-GQDs) and green (G-GQDs) graphene quantum dots. The expression of γH2AX, a marker of DNA double-strand breaks, was determined using flow cytometry. Etoposide (1 µg/mL) is a positive control (PC), 0 is the solvent control (0.2% MilliQ). The distribution of γH2AX APC fluorescence intensity is presented in box plots (95% confidence interval), and three independent experiments were performed. Asterisks (*) indicate a statistically significant difference (two-way ANOVA with uncorrected Fisher’s LSD test) between the solvent control and the exposed cells (** *p* ≤ 0.01)

**Fig. 6:**
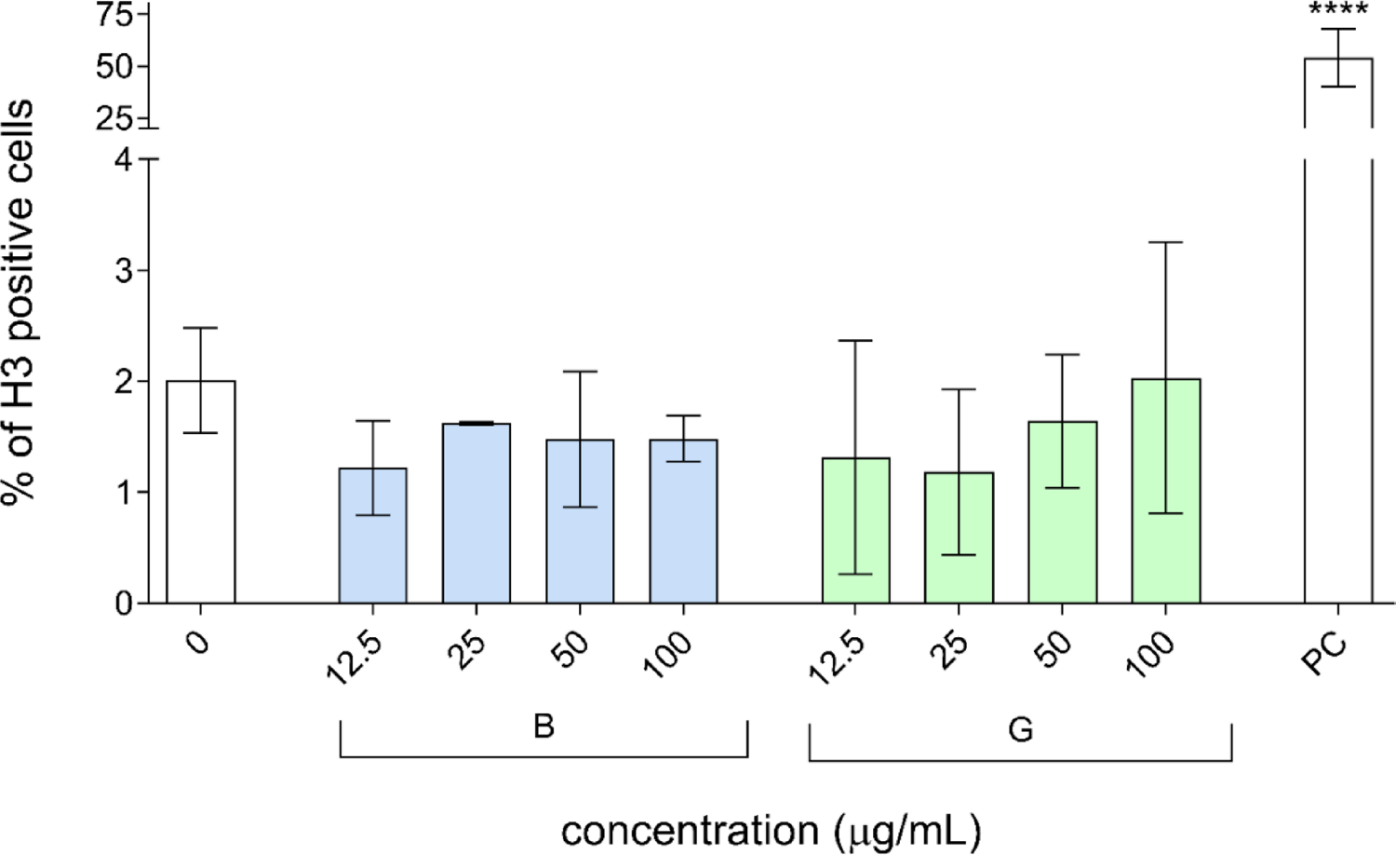
Induction of aneugenic effects in HepG2 cells after 24-hour exposure to blue (B-GQDs) and green (G-GQDs) graphene quantum dots. Phosphorylation of histone H3 (pH3), a marker of aneugenic activity, was determined using flow cytometry. Colchicine (0.2 µg/mL) served as the positive control (PC), 0 is the solvent control (0.2% MilliQ). Results are presented in bar charts as means of three independent experiments. Asterisks (*) indicate a statistically significant difference (ANOVA and Dunnett’s multiple comparison test) between the solvent control and the exposed cells (**** *p* < 0.0001)

 To assess the potential clastogenic and aneugenic effects of B-GQDs and G-GQDs, we evaluated the phosphorylation levels of histone H2AX (γH2AX) and histone H3 (p-H3) in HepG2 spheroids following 24-hour exposure to different concentrations (12.5, 25, 50, and 100 µg/mL, corresponding to 5, 10, 20, and 40 µg/cm^2^) of GQDs. Our results indicate that neither B-GQDs nor G-GQDs induced a significant increase in the phosphorylation of H2AX (Fig. 5) or H3 (Fig. 6) at any of the tested concentrations, suggesting that these GQDs do not cause detectable DNA DSBs or interfere with mitotic progression under the experimental conditions. However, results from the comet assay revealed that at concentrations above 25 µg/mL, both GQDs types induced a significant, dose-dependent increase in primary DNA damage, predominantly in the form of single-strand breaks (SSBs). This suggests that while GQDs do not cause clastogenic damage associated with DSBs, they do induce primary DNA lesions, likely through oxidative stress or direct interaction with DNA. This interpretation aligns with previous studies reporting that GQDs primarily induce SSBs and oxidative DNA damage rather than DSBs, depending on their physicochemical properties, exposure conditions, and cellular responses (Magdolenova et al. 2014; Fadeel et al. 2018). Given that SSBs can be efficiently repaired by base excision repair (BER) or other repair pathways, their accumulation does not necessarily trigger H2AX phosphorylation unless converted to DSBs during replication or repair failure. Similarly, the lack of p-H3 activation suggests that B-GQDs and G-GQDs do not exert aneugenic effects by disrupting mitotic spindle assembly or chromosome segregation. Overall, these results indicate that the genotoxic potential of B-GQDs and G-GQDs is primarily linked to DNA integrity, such as DNA strand breaks, rather than errors in chromosome segregation, known as chromosomal missegregation, which can lead to aneuploidy.

The obtained results contribute to the growing body of evidence required for the safety assessment of graphene-based nanomaterials, including graphene quantum dots (GQDs). Previous studies have highlighted that comprehensive physicochemical characterization, combined with sensitive toxicological endpoints, is essential for robust human health and environmental risk assessment of graphene-derived materials (Fadeel et al. 2018). In this context, the absence of clastogenic and aneugenic effects under acute, non-cytotoxic exposure conditions suggests that these endpoints may not represent the most sensitive indicators of early genotoxic effects induced by GQDs. In contrast, the observed induction of DNA single-strand breaks underscores the relevance of primary DNA damage as a critical endpoint in the regulatory evaluation of graphene-based materials (Domenech et al. 2022). Although the present data are insufficient to establish exposure limits or safety thresholds, they clearly emphasize the importance of including sub-lethal genotoxic endpoints in hazard identification and risk assessment frameworks.

## Conclusion

Taken together, our results demonstrate that, under the tested experimental conditions and non-cytotoxic concentrations used, both B-GQDs and G-GQDs do not induce clastogenic or aneugenic effects in HepG2 spheroids, as shown by the absence of γH2AX and p-H3 phosphorylation. However, both materials induced a clear, dose-dependent increase in DNA single-strand breaks (SSBs), indicating sub-lethal genotoxic stress despite the lack of DNA double-strand breaks (DSBs) or mitotic disruption. These findings suggest that the genotoxic potential of GQDs is primarily associated with primary DNA lesions rather than structural chromosomal damage or errors in chromosome segregation, but limited to the tested concentration range, exposure duration, and the single 3D HepG2 spheroid model used in this study. The observed primary DNA damage is likely linked to oxidative stress, impaired DNA repair pathways, or replication stress—mechanisms that warrant further investigation. Future investigations should focus on characterizing oxidative stress responses, assessing DNA repair kinetics, and evaluating replication fidelity to unravel the molecular basis of GQDs-induced genotoxicity. To fully understand the genotoxic risks posed by GQDs, it will be critical to complement these findings with broader genomic instability assays, such as the micronucleus test, and to conduct long-term exposure studies under physiologically relevant conditions. In addition, evaluating inflammatory and secondary stress pathways will help clarify whether chronic or indirect mechanisms contribute to long-term genotoxic effects. These studies, along with further assessment of oxidative stress markers and DNA repair dynamics, are essential to fully understand the underlying mechanisms of GQDs-induced DNA damage. Together, these efforts will strengthen the mechanistic basis for risk assessment of GQDs and support the safe design, optimisation, and application of graphene-based nanomaterials in biomedical and environmental contexts. From a regulatory perspective, the detection of sub-lethal DNA damage highlights the relevance of primary DNA damage endpoints for the hazard characterization of GQDs, particularly in occupational and biomedical exposure scenarios, even in the absence of overt cytotoxicity or chromosomal alterations.

## Data Availability

All data supporting the findings of this study are included within the article and/or its supplementary materials. All raw datasets generated during the study are available from the corresponding author request and Zenodo (comet assay data—10.5281/zenodo.18375188, and flow cytometry data—10.5281/zenodo.18415123).
